# Crystal structure of chlorido­bis­[(1,2,5,6-η)-cyclo­octa-1,5-diene]iridium(I)

**DOI:** 10.1107/S2056989017000809

**Published:** 2017-01-27

**Authors:** A. K. Fazlur Rahman, Miles Wilklow-Marnell, William W. Brennessel, William D. Jones

**Affiliations:** a Department of Chemistry, University of Rochester, Rochester, NY 14627, USA

**Keywords:** crystal structure, iridium, 1,5-cyclo­octa­diene, five-coordinate complex

## Abstract

The title complex, [IrCl(C_8_H_12_)_2_], was synthesized directly from the reaction of IrCl_3_·3H_2_O with a large excess of cod (cod = cyclo­octa-1,5-diene) in alcoholic solvent. Large yellow needles were obtained by the slow cooling of a hot solution. Based on the positions of the chloride ligand and the mid-points of the four C=C bonds, the mol­ecule adopts a five-coordinate geometry that is midway between square pyramidal and trigonal bipyramidal. The material crystallizes in the ortho­rhom­bic space group *Pbca* with one mol­ecule per asymmetric unit in a general position and shows no significant inter­molecular inter­actions. Individual mol­ecules are aligned along [010], and these rows form a pseudo-hexa­gonal packing arrangement.

## Abstract 2

A direct synthesis of monomeric IrCl(1,5-cod)_2_ (1,5-cod = cyclo­octa-1,5-diene) from IrCl_3_·3H_2_O and its crystal structure are presented. The mol­ecule’s shape is midway between square pyramidal and trigonal bipyramidal.

## Chemical context   

**Figure 1:**
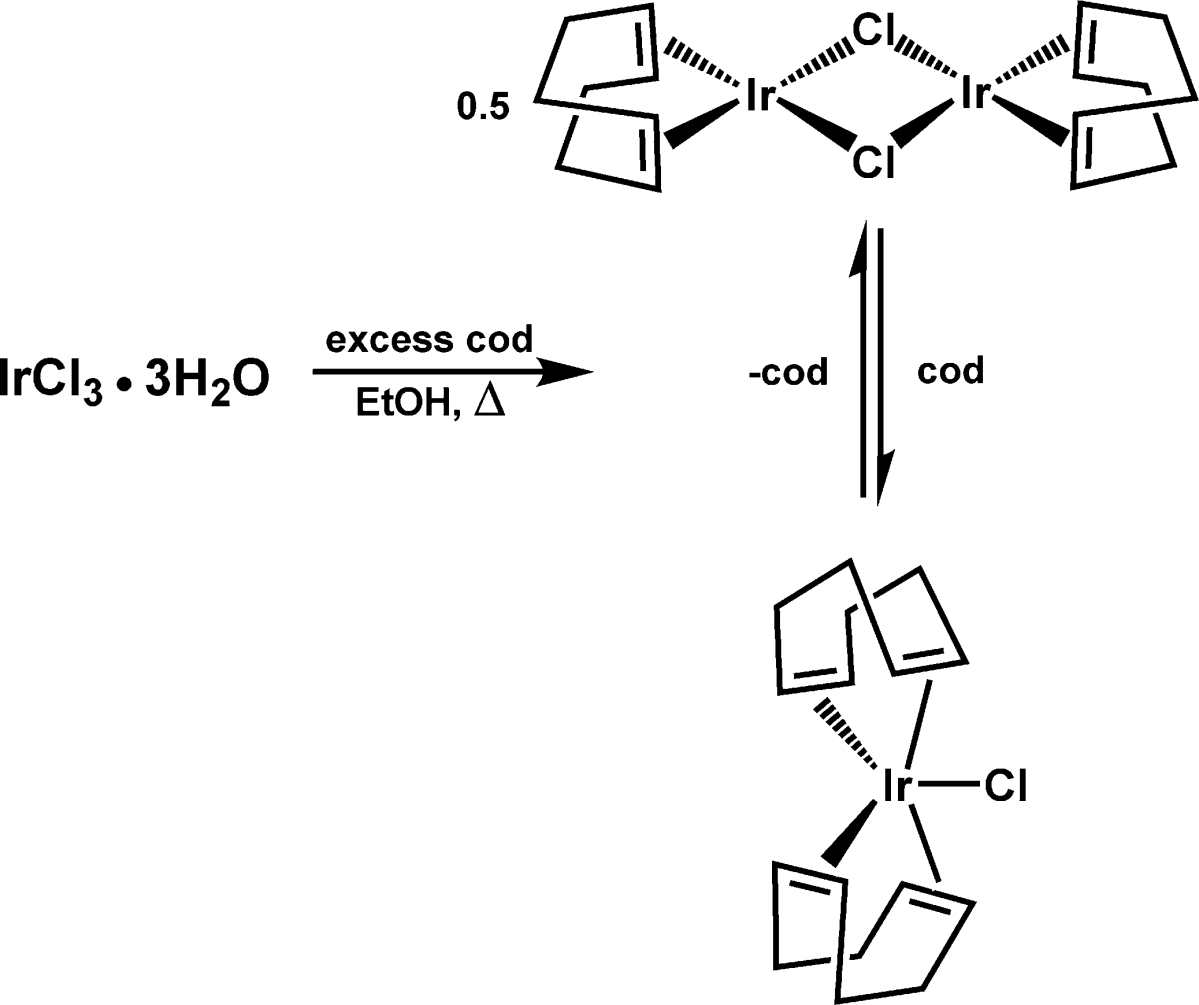
Reaction scheme showing the formation of the mixture of [Ir(cod)(μ-Cl)]_2_ and IrCl(cod)_2_. As the ethanol is removed under vacuum the solution becomes rich in cod, which drives the formation of IrCl(cod)_2_. Loss of cod regenerates the dimer.

First reported in 1966 (Winkhaus & Singer, 1966 ▸) [Ir(cod)(*μ*-Cl)]_2_ (cod = 1,5-cyclo­octa­diene, C_8_H_12_) is perhaps the most common organometallic precursor used in the synthesis of a variety of organoiridium compounds (Leigh & Richards, 1982 ▸). [Ir(cod)(*μ*-Cl)]_2_ can be prepared using either Na_2_IrCl_6_·6H_2_O or IrCl_3_·3H_2_O as the metal-containing precursor (Herde *et al.*, 1974 ▸). A few years later it was reported that a cyclo­octene-ligated dimer [Ir(C_8_H_14_)_2_(μ-Cl)]_2_ had been synthesized from the reaction of ammonium hexa­chlorido­iridiate(III) hydrate, (NH_4_)_3_IrCl_6_·H_2_O, with cyclo­octene in a mixture of 2-propanol and water (Onderdelinden & van der Ent, 1972 ▸). In all three cases, Ir^IV^ or Ir^III^ is reduced to Ir^I^ by oxidation of the alcoholic solvent. Upon suspension in pure cod, [Ir(C_8_H_14_)_2_(*μ*-Cl)]_2_ reacted to form mononuclear IrCl(cod)_2_, which was then characterized by infra-red spectroscopy and elemental analysis (Onderdelinden & van der Ent, 1972 ▸). Analogous to thermally unstable IrCl(C_2_H_4_)_4_, which releases ethyl­ene to form the (slightly) more stable dimer [Ir(C_2_H_4_)_2_(*μ*-Cl)]_2_ (Onderdelinden & van der Ent, 1972 ▸), IrCl(cod)_2_ readily generates stable [Ir(cod)(*μ*-Cl)]_2_ with the loss of one equivalent of cod per iridium. We have found that if Herde’s preparation using IrCl_3_·3H_2_O is carried out with a large excess of cod (10 ×), the product isolated after removal of the alcoholic solvent is IrCl(cod)_2_ (Fig. 1 ▸). This was apparent as the red–orange reaction mixture, which contained a mixture of red [Ir(cod)(*μ*-Cl)]_2_ and yellow IrCl(cod)_2_, became pale yellow. Recrystallization from refluxing methanol/cod (7:1, *v*:*v*) followed by cooling produced yellow needles of IrCl(cod)_2_ suitable for diffraction studies.

Herein we report the isolation and results of the single structure determination of mononuclear IrCl(cod)_2_ and compare it to related Ir*X*(diene)_2_ (*X* = Cl, SnMe_3_, SnCl_3_) complexes.
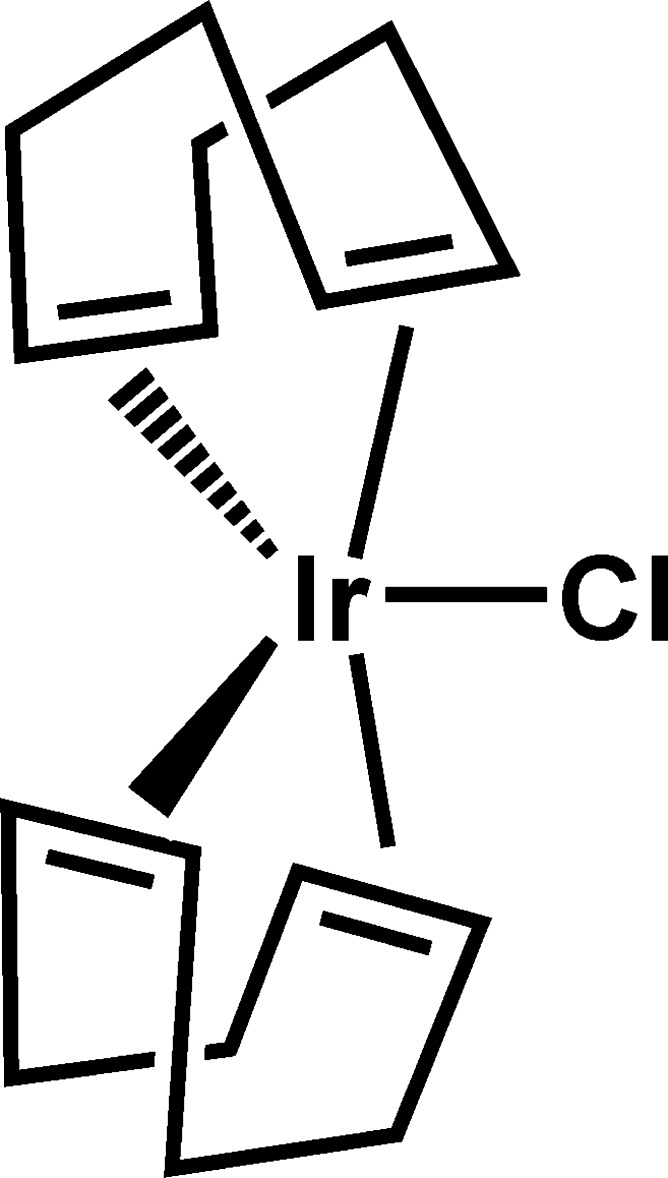


## Structural commentary   

**Figure 2:**
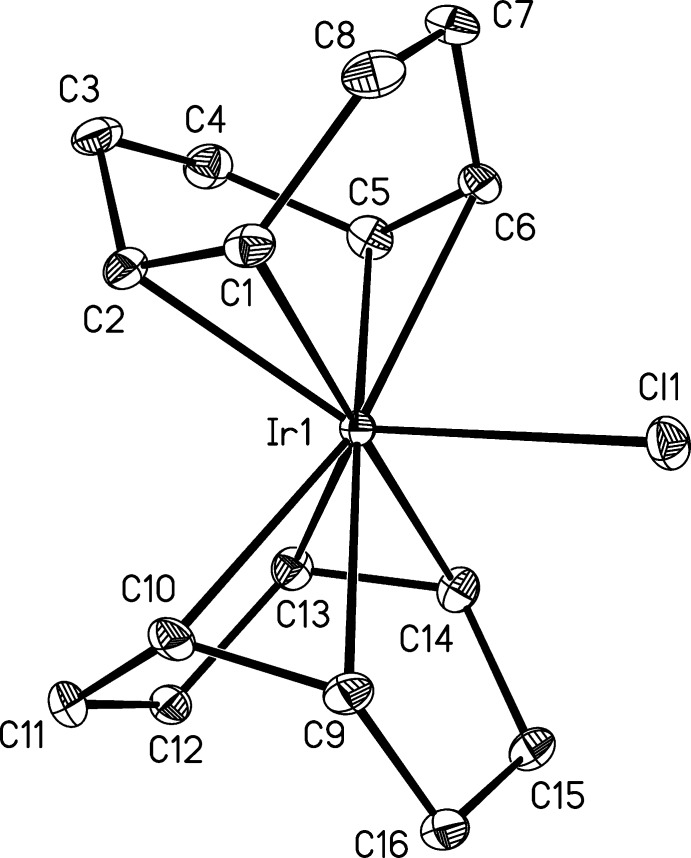
The mol­ecular structure of IrCl(cod)_2_, with displacement ellipsoids drawn at the 50% probability level.

Our single-crystal X-ray diffraction study confirmed the mol­ecule to be mononuclear IrCl(cod)_2_, in which the two cod ligands are bound in an η^2^:η^2^ fashion (Fig. 2 ▸). The material crystallizes in the ortho­rhom­bic space group *Pbca*, with one mol­ecule per asymmetric unit in a general position. The five-coordinate complex adopts a geometry that is midway between square pyramidal (SP) and trigonal bipyramidal (TBP), with a *τ*_5_ parameter of 0.52 (Addison *et al.*, 1984 ▸), calculated using the mid-points of the C=C double bonds and the axial chlorido ligand. The elongation of the cod double bonds (Table 1 ▸) compared to those of non-coordinating cod, 1.333 (4) and 1.334 (4) Å (Byrn *et al.*, 1990 ▸), or to that of free ethyl­ene, 1.333 Å (Lide, 2002–2003 ▸), is consistent with back donation to the π* orbitals from a low-valent iridium atom, formally Ir^I^. The elongations are asymmetric, with one double bond from each cod ligand being larger than the other by 0.048 (6) and 0.029 (6) Å, respectively, for cod ligands C1—C8 and C9—C16. Likewise the distances between Ir and the mid-points of the C=C bonds also show this asymmetry with two shorter distances, Ir—(C1/C2) = 2.047 (4) and Ir—(C9/C10) = 2.069 (4) Å, and two longer distances, Ir—(C5/C6) = 2.138 (4) and Ir—(C13/C14) = 2.141 (4) Å (Table 2 ▸). This is likely due to its inter­mediacy between the geometric extremes of SP and TBP. Ideal SP geometry (*τ*_5_ = 0) would have very similar Ir–mid-point(C=C) distances as they would involve the same metal and ligand orbitals, while ideal TBP geometry (*τ*_5_ = 1) would involve different orbitals, dependent upon on whether the ligand’s C=C bond lay in an axial or an equatorial position. We see the former (SP) in Ir(SnCl_3_)(nbd)_2_ (nbd = norbornadiene; Malosh *et al.*, 2013 ▸), for which *τ*_5_ = 0.06 and the Ir–mid-point(C=C) distances are similar, ranging from 2.067 (4) to 2.089 (4) Å. An example towards TBP is found in [IrCl(cod)(CC*)]^+^ (CC* = [(η^5^-C_5_H_5_)Fe(η^6^-(1,1-di(2-propen­yl)-3-buten­yl)benzene)]; Marcén *et al.*, 2002 ▸), for which *τ*_5_ = 0.76.

## Supra­molecular features   

**Figure 4:**
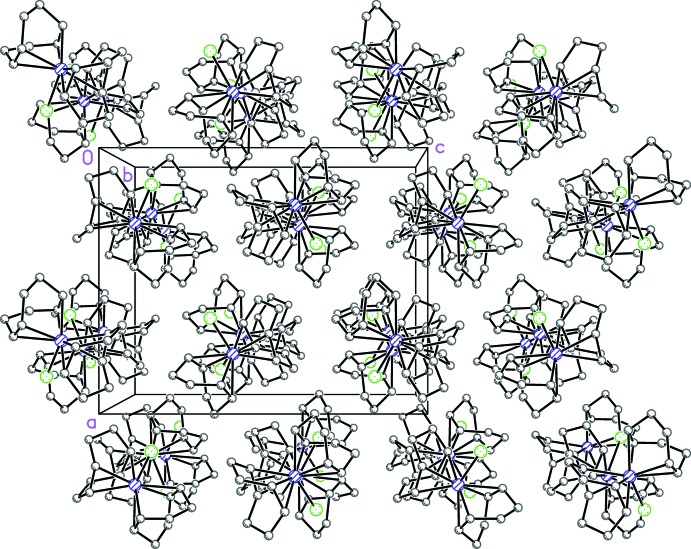
Pseudo-hexa­gonal arrangement of rows of mol­ecules aligned along [010].

Although there are no significant inter­molecular inter­actions, the packing has adopted a supra­molecular arrangement. Individual mol­ecules are aligned in columns parallel to [010], which are then arranged in an overall pseudo-hexa­gonal packing (Fig. 4 ▸).

## Database survey   

A survey of the Cambridge Structural Database (CSD, Version 5.38, update No. 1, November 2016, Groom *et al.*, 2016 ▸) revealed just a few related five-coordinate iridium complexes with four unconjugated substituted ethyl­ene ligands and a halido or stannato ligand in the fifth coordination site: Ir(SnCl_3_)(cod)_2_ (CSD refcode COIRSN; Porta *et al.*, 1967 ▸), Ir(SnMe_3_)(cod)_2_ (refcode DIVPAB), Ir(SnCl_3_)(nbd)_2_ (refcode DIVPIJ), Ir(SnMe_3_)(nbd)_2_ (refcode DIVNUT); Malosh *et al.*, 2013 ▸), and [IrCl(cod)(CC*)]^+^ (refcode PUYCOB; Marcén *et al.*, 2002 ▸). A report on the structure of IrCl(C_2_H_4_)_4_ exists, but no positional parameters were given (van der Ent & van Soest, 1970 ▸), which is unfortunate because a comparison of this species with IrCl(cod)_2_ would ostensibly show how the bite-angle restrictions imposed by the cod rings affect the overall geometry. The geometries of the two tin-containing compounds with cod are closely related to that of the title complex. Both Ir(SnCl_3_)(cod)_2_ and Ir(SnMe_3_)(cod)_2_ exhibit the same long–short variation of the Ir–mid-point(C=C) bond lengths within each cod ligand and have similar *τ*_5_ parameters of 0.53 and 0.55, respectively (Table 2 ▸). Malosh and coworkers concluded that the bulk of the cod ligands relative to that of the nbd ligands was responsible for the geometric distortion from SP geometry, specifically due to CH_2_⋯Me and CH_2_⋯Cl repulsions (Malosh *et al.*, 2013 ▸). And indeed the two nbd complexes have near-perfect SP *τ*_5_ values of 0.10 and 0.06. In complex [IrCl(cod)(CC*)]^+^, the non-cod diene is part of a 1,1-di(2-propen­yl)-3-buten­yl)benzene unit that is η^6^-coordinating to an [Fe(C_5_H_5_)]^+^ cationic fragment. The penta­coordinated saturated (18 electron) iridium atom approaches a TBP geometry more than the other complexes mentioned (*τ*_5_ = 0.76), with the two apical positions being occupied by one C=C bond of the cod ligand and the chlorido ligand. The angles in the equatorial plane range between 109.73 (17) and 126.61 (16)°. The restriction of the cod ligand with its bite angle of 84.9 (2)° prevents the structure from ever achieving perfect TBP geometry, and this holds more so for structures with nbd ligands whose bite angles are even more acute. The Ir–mid-point(C=C) bond lengths differ, showing significantly longer bond lengths to the allylic C=C centroids [avg. 2.144 (11) Å] than to the cod C=C diolefin centroids [avg. 2.052 (11) Å]. The terminal Ir—Cl distance in IrCl(cod)_2_ of 2.5573 (8) Å is longer than all of the 214 structures with five-coordinate iridium in the CSD containing an IrCl(η^2^:η^2^-cod) fragment (avg. 2.368 Å), which may be attributable to its tendency to form the well-known stable cationic complex, [Ir(cod)_2_]^+^, whose structure (refcode TUQWOS) displays the anti­cipated *d* ^8^ square-planar geometry with [BAr^F^]^−^ {tetra­kis­[3,5-bis­(tri­fluoro­meth­yl)phen­yl]borate} as the non-coordinating anion (Woodmansee *et al.*, 2010 ▸).

## Synthesis and crystallization   

All operations and routine manipulations were performed under a nitro­gen atmosphere, either on a high-vacuum line using modified Schlenk techniques or in a Vacuum Atmospheres Company Dri-Lab. A preparation of IrCl(cod)_2_ *via* a cyclo­octene-ligated dimer has been reported previously (Onderdelinden & van der Ent, 1972 ▸).

A two-necked round-bottom flask was charged with IrCl_3_·3H_2_O (6.0 g, 0.017 mol) and cod (20 g, 0.18 mol) in 80 ml of ethanol under nitro­gen. The reaction mixture was refluxed for 24 h, followed by removal of the solvent under vacuum. As the ethanol evaporated, the red–orange solution became more yellow as the cod concentration increased, leading to the isolation of a yellow solid (5.32 g, 70.5%). The product was recrystallized by refluxing in a mixture 35 ml of methanol and 5 ml of cod, followed by cooling to obtain shiny yellow needles of IrCl(cod)_2_ (5.06 g, 67.0%).

## Refinement   

Crystal data, data collection and structure refinement details are summarized in Table 3 ▸. H atoms were treated in the riding-model approximation, with C(methine)—H = 1.00 Å, *C*(methyl­ene)—H = 0.99 Å, and with *U*_iso_(H) = 1.2*U*_eq_(C). The maximum and minimum electron densities are found 1.09 and 0.55 Å, respectively, from the iridium atom.

## Supplementary Material

Crystal structure: contains datablock(s) I, global. DOI: 10.1107/S2056989017000809/wm5359sup1.cif

Structure factors: contains datablock(s) I. DOI: 10.1107/S2056989017000809/wm5359Isup2.hkl

CCDC reference: 1527918

Additional supporting information:  crystallographic information; 3D view; checkCIF report

## Figures and Tables

**Figure 3:**
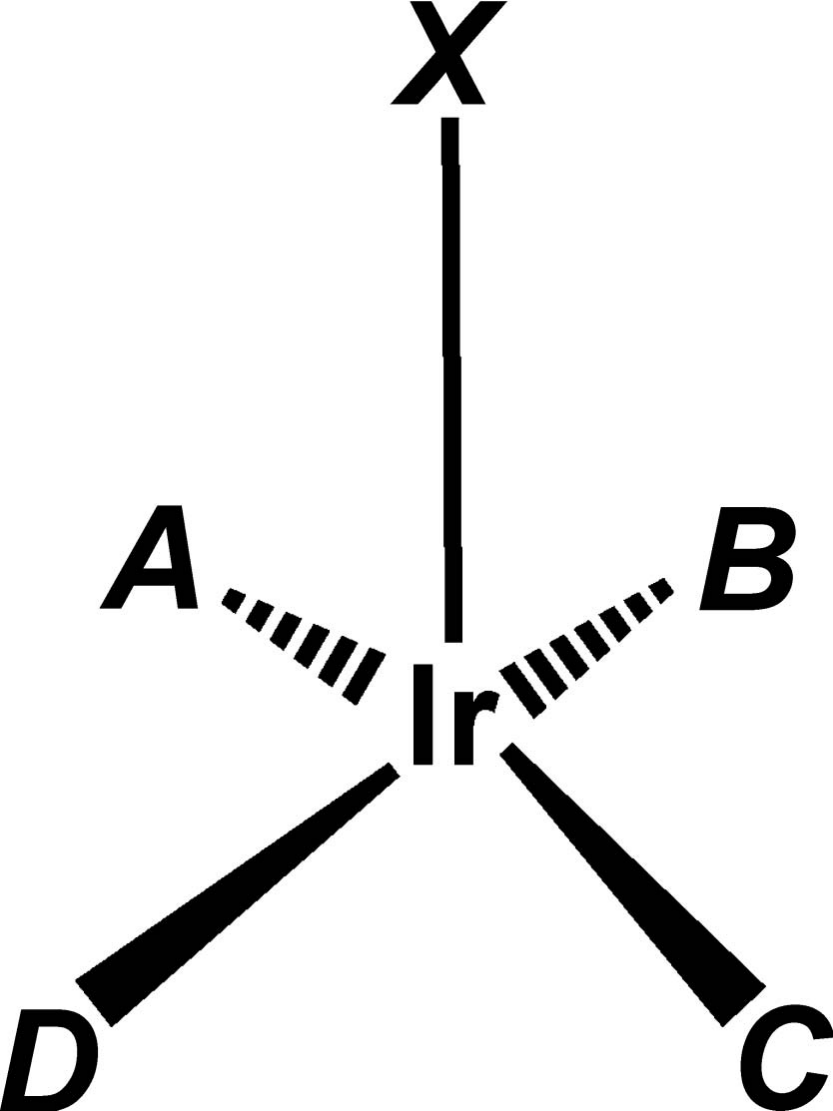
Lettering scheme used for bonds in Table 2 ▸. Letters *A*–*D* are the mid-points of the C=C bonds. In cases of cyclo­dienes, consecutive letters *A*, *B* and/or *C*, *D* are on the same ligand; axial ligand *X* is Sn*R*_3_ or Cl.

**Table 1:** Selected bond lengths (Å)

Ir1—Cl1	2.5573 (8)	C9—C10	1.418 (5)
C1—C2	1.437 (5)	C13—C14	1.389 (4)
C5—C6	1.389 (4)		

**Table 2:** Comparison of bond lengths (Å) and τ_5_ parameters for selected five-coordinate Ir complexes containing four substituted ethyl­ene ligands and a terminal chlorido or stannato ligand, according to the labeling in Fig. 3 ▸

Feature	IrCl(cod)_2_	COIRSN^*a*^	DIVPAB^*b*^	DIVNUT^*c*^	DIVPIJ^*d*^	PUYCOB^*e*^
*M*—*X* ^*f*^	2.5573 (8)	2.642 (2)	2.7090 (4)	2.6606 (4)	2.5850 (9)	2.3883 (15)
*M*—[*A*]	2.047 (4)	2.068 (31)	2.062 (4)	2.067 (4)	2.101 (5)	2.048 (8)
*M*—[*B*]	2.138 (4)	2.135 (26)	2.114 (4)	2.089 (4)	2.119 (5)	2.057 (8)
*M*—[*C*]	2.069 (4)	2.053 (34)	2.069 (4)	2.076 (4)	2.104 (5)	2.118 (8)
*M*—[*D*]	2.141 (4)	2.134 (24)	2.126 (4)	2.089 (4)	2.109 (5)	2.170 (8)
						
C=C[*A*]	1.437 (5)	1.320 (40)	1.425 (5)	1.408 (6)	1.415(8)	1.395 (9)
C=C[*B*]	1.389 (4)	1.450 (44)	1.416 (5)	1.415 (7)	1.389 (7)	1.372 (12)
C=C[*C*]	1.418 (5)	1.361 (44)	1.411 (5)	1.400 (7)	1.394 (7)	1.393 (8)
C=C[*D*]	1.389 (4)	1.375 (41)	1.407 (5)	1.423 (7)	1.404 (8)	1.386 (9)
						
τ_5_^*g*^	0.52	0.53	0.55	0.10	0.06	0.76

Notes: (*a*) Ir(SnCl_3_)(cod)_2_ (Porta *et al.*, 1967 ▸); (*b*) Ir(SnMe_3_)(cod)_2_ (Malosh *et al.*, 2013 ▸); (*c*) Ir(SnMe_3_)(nbd)_2_ (Malosh *et al.*, 2013 ▸); (*d*) Ir(SnCl_3_)(nbd)_2_ (Malosh *et al.*, 2013 ▸); (*e*) [IrCl(cod)(CC*)]^+^ (Marcén *et al.*, 2002 ▸); (*f*) *X* = Sn or Cl; (*g*) Addison *et al.* (1984 ▸).

**Table 3:** Experimental details

Crystal data	
Chemical formula	[IrCl(C_8_H_12_)_2_]
*M*_r_	444.00
Crystal system, space group	Orthorhombic, *P* *b* *c* *a*
Temperature (K)	100
*a*, *b*, *c* (Å)	12.8756 (8), 13.3719 (8), 15.9033 (10)
*V* (Å^3^)	2738.1 (3)
*Z*	8
Radiation type	Mo *K*α
μ (mm^−1^)	9.93
Crystal size (mm)	0.24 × 0.20 × 0.20

Data collection	
Diffractometer	Bruker SMART APEXII CCD platform
Absorption correction	Multi-scan (*SADABS*; Krause *et al.*, 2015 ▸)
*T*_min_, *T*_max_	0.173, 0.278
No. of measured, independent and observed [*I* > 2σ(*I*)] reflections	83189, 7755, 5394
*R*_int_	0.112
(sin θ/λ)_max_ (Å^−1^)	0.883

Refinement	
*R*[*F* ^2^ > 2σ(*F* ^2^)], *wR*(*F* ^2^), *S*	0.035, 0.072, 1.01
No. of reflections	7755
No. of parameters	163
H-atom treatment	H-atom parameters constrained
Δρ_max_, Δρ_min_ (e Å^−3^)	1.34, −1.86

Computer programs: *APEX3* and *SAINT* (Bruker, 2016 ▸), *SHELXT2014* (Sheldrick, 2015*a* ▸), *SHELXL2016* (Sheldrick, 2015*b* ▸) and *SHELXTL* (Sheldrick, 2008 ▸).

## supplementary crystallographic information

### Crystal data

**Table:**

[IrCl(C_8_H_12_)_2_]	*D*_x_ = 2.154 Mg m^−^^3^
*M**_r_* = 444.00	Mo *K*α radiation, λ = 0.71073 Å
Orthorhombic, *P**b**c**a*	Cell parameters from 5121 reflections
*a* = 12.8756 (8) Å	θ = 2.5–29.0°
*b* = 13.3719 (8) Å	µ = 9.93 mm^−^^1^
*c* = 15.9033 (10) Å	*T* = 100 K
*V* = 2738.1 (3) Å^3^	Block, yellow
*Z* = 8	0.24 × 0.20 × 0.20 mm
*F*(000) = 1712	

### Data collection

**Table:**

Bruker SMART APEXII CCD platform diffractometer	5394 reflections with *I* > 2σ(*I*)
Radiation source: fine-focus sealed tube	*R*_int_ = 0.112
ω scans	θ_max_ = 38.9°, θ_min_ = 2.5°
Absorption correction: multi-scan (*SADABS*; Krause *et al.*, 2015)	*h* = −22→22
*T*_min_ = 0.173, *T*_max_ = 0.278	*k* = −23→23
83189 measured reflections	*l* = −27→27
7755 independent reflections	

### Refinement

**Table:**

Refinement on *F*^2^	Primary atom site location: structure-invariant direct methods
Least-squares matrix: full	Secondary atom site location: difference Fourier map
*R*[*F*^2^ > 2σ(*F*^2^)] = 0.035	Hydrogen site location: inferred from neighbouring sites
*wR*(*F*^2^) = 0.072	H-atom parameters constrained
*S* = 1.01	*w* = 1/[σ^2^(*F*_o_^2^) + (0.0214*P*)^2^] where *P* = (*F*_o_^2^ + 2*F*_c_^2^)/3
7755 reflections	(Δ/σ)_max_ = 0.001
163 parameters	Δρ_max_ = 1.34 e Å^−^^3^
0 restraints	Δρ_min_ = −1.86 e Å^−^^3^

### Special details

**Table:**

Geometry. All esds (except the esd in the dihedral angle between two l.s. planes) are estimated using the full covariance matrix. The cell esds are taken into account individually in the estimation of esds in distances, angles and torsion angles; correlations between esds in cell parameters are only used when they are defined by crystal symmetry. An approximate (isotropic) treatment of cell esds is used for estimating esds involving l.s. planes.

### Fractional atomic coordinates and isotropic or equivalent isotropic displacement parameters (Å^2^)

**Table:**

	*x*	*y*	*z*	*U*_iso_*/*U*_eq_	
Ir1	0.27908 (2)	0.39530 (2)	0.59862 (2)	0.00769 (3)	
Cl1	0.13983 (6)	0.50996 (6)	0.66069 (5)	0.01377 (14)	
C1	0.1656 (3)	0.2813 (2)	0.5723 (2)	0.0116 (5)	
H1A	0.145496	0.241248	0.622824	0.014*	
C2	0.2626 (2)	0.2509 (2)	0.5357 (2)	0.0123 (6)	
H2A	0.296862	0.194607	0.566472	0.015*	
C3	0.2855 (3)	0.2514 (3)	0.4419 (2)	0.0143 (6)	
H3A	0.219685	0.241850	0.410691	0.017*	
H3B	0.331572	0.194314	0.428387	0.017*	
C4	0.3373 (3)	0.3486 (3)	0.4120 (2)	0.0157 (6)	
H4A	0.413620	0.341604	0.416347	0.019*	
H4B	0.319781	0.360032	0.352151	0.019*	
C5	0.3028 (3)	0.4380 (2)	0.4632 (2)	0.0126 (6)	
H5A	0.347039	0.498657	0.455193	0.015*	
C6	0.2005 (2)	0.4593 (2)	0.4845 (2)	0.0114 (6)	
H6A	0.186019	0.532519	0.489222	0.014*	
C7	0.1063 (3)	0.3986 (2)	0.4565 (2)	0.0142 (6)	
H7A	0.122928	0.363524	0.403371	0.017*	
H7B	0.047558	0.444498	0.445506	0.017*	
C8	0.0736 (3)	0.3211 (3)	0.5234 (2)	0.0157 (6)	
H8A	0.023939	0.352582	0.562866	0.019*	
H8B	0.037702	0.264779	0.495361	0.019*	
C9	0.4463 (2)	0.4276 (2)	0.5992 (2)	0.0129 (5)	
H9A	0.476894	0.435608	0.541763	0.015*	
C10	0.3907 (2)	0.5127 (2)	0.6275 (2)	0.0127 (6)	
H10A	0.390267	0.569889	0.587016	0.015*	
C11	0.3863 (3)	0.5434 (2)	0.7186 (2)	0.0145 (6)	
H11A	0.334721	0.597717	0.724955	0.017*	
H11B	0.454933	0.570368	0.735239	0.017*	
C12	0.3573 (3)	0.4571 (2)	0.7786 (2)	0.0143 (6)	
H12A	0.421967	0.428574	0.802348	0.017*	
H12B	0.316321	0.484671	0.825894	0.017*	
C13	0.2957 (3)	0.3738 (2)	0.7378 (2)	0.0122 (6)	
H13A	0.228508	0.359314	0.766834	0.015*	
C14	0.3391 (3)	0.2906 (2)	0.6990 (2)	0.0121 (5)	
H14A	0.296863	0.228253	0.705481	0.014*	
C15	0.4533 (3)	0.2717 (2)	0.6889 (2)	0.0143 (6)	
H15A	0.463587	0.216505	0.648278	0.017*	
H15B	0.482493	0.250336	0.743567	0.017*	
C16	0.5120 (2)	0.3639 (2)	0.6581 (2)	0.0132 (6)	
H16A	0.533137	0.404768	0.707119	0.016*	
H16B	0.575745	0.342441	0.628395	0.016*	

### Atomic displacement parameters (Å^2^)

**Table:**

	*U*^11^	*U*^22^	*U*^33^	*U*^12^	*U*^13^	*U*^23^
Ir1	0.00792 (5)	0.00752 (5)	0.00763 (5)	−0.00044 (4)	−0.00014 (4)	0.00038 (4)
Cl1	0.0124 (3)	0.0145 (3)	0.0144 (4)	0.0035 (3)	0.0018 (3)	0.0005 (3)
C1	0.0128 (13)	0.0107 (13)	0.0111 (13)	−0.0039 (11)	−0.0013 (11)	0.0010 (10)
C2	0.0142 (14)	0.0104 (13)	0.0123 (14)	−0.0015 (11)	−0.0020 (11)	−0.0017 (11)
C3	0.0144 (14)	0.0176 (14)	0.0109 (14)	−0.0007 (12)	−0.0019 (12)	−0.0048 (11)
C4	0.0157 (14)	0.0192 (15)	0.0124 (15)	0.0007 (12)	0.0012 (12)	−0.0017 (12)
C5	0.0131 (13)	0.0136 (14)	0.0112 (14)	−0.0016 (11)	0.0016 (11)	0.0035 (11)
C6	0.0152 (14)	0.0103 (13)	0.0088 (13)	0.0003 (10)	−0.0017 (11)	0.0034 (10)
C7	0.0114 (13)	0.0173 (14)	0.0138 (14)	−0.0011 (12)	−0.0040 (11)	0.0007 (12)
C8	0.0131 (14)	0.0182 (15)	0.0157 (16)	−0.0057 (12)	−0.0012 (12)	−0.0014 (12)
C9	0.0117 (13)	0.0132 (13)	0.0137 (14)	0.0000 (10)	0.0007 (12)	−0.0007 (12)
C10	0.0136 (13)	0.0109 (13)	0.0137 (15)	−0.0019 (11)	0.0017 (12)	−0.0002 (11)
C11	0.0163 (14)	0.0125 (14)	0.0148 (16)	−0.0018 (11)	−0.0025 (12)	−0.0040 (11)
C12	0.0153 (14)	0.0170 (15)	0.0106 (14)	0.0025 (12)	−0.0018 (11)	−0.0025 (11)
C13	0.0145 (14)	0.0146 (14)	0.0076 (13)	0.0001 (11)	0.0011 (11)	−0.0005 (10)
C14	0.0159 (14)	0.0109 (13)	0.0094 (13)	−0.0004 (11)	−0.0012 (12)	0.0043 (10)
C15	0.0150 (14)	0.0121 (14)	0.0158 (16)	0.0046 (11)	−0.0035 (12)	−0.0004 (11)
C16	0.0106 (13)	0.0153 (14)	0.0137 (15)	0.0026 (11)	−0.0026 (11)	−0.0022 (12)

### Geometric parameters (Å, º)

**Table:**

Ir1—C1	2.152 (3)	C7—H7A	0.9900
Ir1—C10	2.178 (3)	C7—H7B	0.9900
Ir1—C2	2.185 (3)	C8—H8A	0.9900
Ir1—C9	2.196 (3)	C8—H8B	0.9900
Ir1—C13	2.242 (3)	C9—C10	1.418 (5)
Ir1—C6	2.248 (3)	C9—C16	1.522 (5)
Ir1—C5	2.249 (3)	C9—H9A	1.0000
Ir1—C14	2.260 (3)	C10—C11	1.506 (5)
Ir1—Cl1	2.5573 (8)	C10—H10A	1.0000
C1—C2	1.437 (5)	C11—C12	1.543 (5)
C1—C8	1.514 (5)	C11—H11A	0.9900
C1—H1A	1.0000	C11—H11B	0.9900
C2—C3	1.521 (5)	C12—C13	1.514 (5)
C2—H2A	1.0000	C12—H12A	0.9900
C3—C4	1.537 (5)	C12—H12B	0.9900
C3—H3A	0.9900	C13—C14	1.389 (4)
C3—H3B	0.9900	C13—H13A	1.0000
C4—C5	1.512 (5)	C14—C15	1.500 (4)
C4—H4A	0.9900	C14—H14A	1.0000
C4—H4B	0.9900	C15—C16	1.527 (5)
C5—C6	1.389 (4)	C15—H15A	0.9900
C5—H5A	1.0000	C15—H15B	0.9900
C6—C7	1.525 (4)	C16—H16A	0.9900
C6—H6A	1.0000	C16—H16B	0.9900
C7—C8	1.543 (5)		
			
C1—Ir1—C10	178.38 (12)	C5—C6—C7	125.0 (3)
C1—Ir1—C2	38.68 (12)	C5—C6—Ir1	72.04 (18)
C10—Ir1—C2	142.92 (12)	C7—C6—Ir1	113.0 (2)
C1—Ir1—C9	143.67 (12)	C5—C6—H6A	113.3
C10—Ir1—C9	37.84 (12)	C7—C6—H6A	113.3
C2—Ir1—C9	105.71 (12)	Ir1—C6—H6A	113.3
C1—Ir1—C13	99.56 (12)	C6—C7—C8	111.9 (3)
C10—Ir1—C13	79.68 (12)	C6—C7—H7A	109.2
C2—Ir1—C13	110.35 (12)	C8—C7—H7A	109.2
C9—Ir1—C13	85.84 (12)	C6—C7—H7B	109.2
C1—Ir1—C6	78.85 (12)	C8—C7—H7B	109.2
C10—Ir1—C6	101.16 (12)	H7A—C7—H7B	107.9
C2—Ir1—C6	85.57 (12)	C1—C8—C7	112.1 (3)
C9—Ir1—C6	111.72 (12)	C1—C8—H8A	109.2
C13—Ir1—C6	152.77 (12)	C7—C8—H8A	109.2
C1—Ir1—C5	94.89 (12)	C1—C8—H8B	109.2
C10—Ir1—C5	85.98 (12)	C7—C8—H8B	109.2
C2—Ir1—C5	78.39 (12)	H8A—C8—H8B	107.9
C9—Ir1—C5	79.69 (12)	C10—C9—C16	122.2 (3)
C13—Ir1—C5	164.79 (12)	C10—C9—Ir1	70.37 (18)
C6—Ir1—C5	36.00 (11)	C16—C9—Ir1	115.9 (2)
C1—Ir1—C14	86.02 (12)	C10—C9—H9A	113.8
C10—Ir1—C14	94.13 (12)	C16—C9—H9A	113.8
C2—Ir1—C14	78.99 (12)	Ir1—C9—H9A	113.8
C9—Ir1—C14	77.53 (12)	C9—C10—C11	122.9 (3)
C13—Ir1—C14	35.94 (11)	C9—C10—Ir1	71.79 (18)
C6—Ir1—C14	163.81 (12)	C11—C10—Ir1	112.0 (2)
C5—Ir1—C14	141.95 (12)	C9—C10—H10A	114.3
C1—Ir1—Cl1	91.36 (9)	C11—C10—H10A	114.3
C10—Ir1—Cl1	87.07 (9)	Ir1—C10—H10A	114.3
C2—Ir1—Cl1	129.69 (8)	C10—C11—C12	113.6 (3)
C9—Ir1—Cl1	124.61 (9)	C10—C11—H11A	108.8
C13—Ir1—Cl1	76.29 (8)	C12—C11—H11A	108.8
C6—Ir1—Cl1	76.57 (9)	C10—C11—H11B	108.8
C5—Ir1—Cl1	108.22 (9)	C12—C11—H11B	108.8
C14—Ir1—Cl1	109.78 (9)	H11A—C11—H11B	107.7
C2—C1—C8	124.9 (3)	C13—C12—C11	114.3 (3)
C2—C1—Ir1	71.90 (18)	C13—C12—H12A	108.7
C8—C1—Ir1	112.5 (2)	C11—C12—H12A	108.7
C2—C1—H1A	113.5	C13—C12—H12B	108.7
C8—C1—H1A	113.5	C11—C12—H12B	108.7
Ir1—C1—H1A	113.5	H12A—C12—H12B	107.6
C1—C2—C3	124.4 (3)	C14—C13—C12	124.7 (3)
C1—C2—Ir1	69.42 (18)	C14—C13—Ir1	72.71 (18)
C3—C2—Ir1	115.3 (2)	C12—C13—Ir1	112.3 (2)
C1—C2—H2A	113.5	C14—C13—H13A	113.4
C3—C2—H2A	113.5	C12—C13—H13A	113.4
Ir1—C2—H2A	113.5	Ir1—C13—H13A	113.4
C2—C3—C4	113.0 (3)	C13—C14—C15	125.1 (3)
C2—C3—H3A	109.0	C13—C14—Ir1	71.35 (18)
C4—C3—H3A	109.0	C15—C14—Ir1	111.3 (2)
C2—C3—H3B	109.0	C13—C14—H14A	113.8
C4—C3—H3B	109.0	C15—C14—H14A	113.8
H3A—C3—H3B	107.8	Ir1—C14—H14A	113.8
C5—C4—C3	112.0 (3)	C14—C15—C16	112.6 (3)
C5—C4—H4A	109.2	C14—C15—H15A	109.1
C3—C4—H4A	109.2	C16—C15—H15A	109.1
C5—C4—H4B	109.2	C14—C15—H15B	109.1
C3—C4—H4B	109.2	C16—C15—H15B	109.1
H4A—C4—H4B	107.9	H15A—C15—H15B	107.8
C6—C5—C4	124.9 (3)	C9—C16—C15	112.0 (3)
C6—C5—Ir1	71.96 (18)	C9—C16—H16A	109.2
C4—C5—Ir1	110.8 (2)	C15—C16—H16A	109.2
C6—C5—H5A	113.9	C9—C16—H16B	109.2
C4—C5—H5A	113.9	C15—C16—H16B	109.2
Ir1—C5—H5A	113.9	H16A—C16—H16B	107.9
			
C8—C1—C2—C3	−2.0 (5)	C16—C9—C10—C11	−4.0 (5)
Ir1—C1—C2—C3	−107.2 (3)	Ir1—C9—C10—C11	104.9 (3)
C8—C1—C2—Ir1	105.2 (3)	C16—C9—C10—Ir1	−108.9 (3)
C1—C2—C3—C4	93.2 (4)	C9—C10—C11—C12	−49.6 (4)
Ir1—C2—C3—C4	11.7 (4)	Ir1—C10—C11—C12	32.3 (3)
C2—C3—C4—C5	−31.5 (4)	C10—C11—C12—C13	−24.8 (4)
C3—C4—C5—C6	−46.1 (4)	C11—C12—C13—C14	89.4 (4)
C3—C4—C5—Ir1	35.8 (3)	C11—C12—C13—Ir1	5.6 (3)
C4—C5—C6—C7	−2.8 (5)	C12—C13—C14—C15	−2.1 (5)
Ir1—C5—C6—C7	−106.0 (3)	Ir1—C13—C14—C15	103.4 (3)
C4—C5—C6—Ir1	103.2 (3)	C12—C13—C14—Ir1	−105.5 (3)
C5—C6—C7—C8	95.7 (4)	C13—C14—C15—C16	−45.8 (4)
Ir1—C6—C7—C8	12.1 (3)	Ir1—C14—C15—C16	35.9 (3)
C2—C1—C8—C7	−44.6 (4)	C10—C9—C16—C15	97.1 (4)
Ir1—C1—C8—C7	38.5 (3)	Ir1—C9—C16—C15	14.8 (4)
C6—C7—C8—C1	−32.5 (4)	C14—C15—C16—C9	−33.3 (4)

## References

[bb1] Addison, A. W., Rao, T. N., Reedijk, J., van Rijn, J. & Verschoor, G. C. (1984). *J. Chem. Soc. Dalton Trans.* pp. 1349–1356.

[bb2] Bruker (2016). *APEX3* and *SAINT*, Bruker AXS, Inc., Madison, Wisconsin, USA.

[bb3] Byrn, M. P., Curtis, C. J., Khan, S. I., Sawin, P. A., Tsurumi, R. & Strouse, C. E. (1990). *J. Am. Chem. Soc.* **112**, 1865–1874.

[bb4] Ent, A. van der & van Soest, T. C. (1970). *Chem. Commun.* pp. 225–226.

[bb5] Groom, C. R., Bruno, I. J., Lightfoot, M. P. & Ward, S. C. (2016). *Acta Cryst.* B**72**, 171–179. 10.1107/S2052520616003954 PMC4822653 27048719

[bb6] Herde, J. L., Lambert, J. C. & Senoff, C. V. (1974). *Inorg. Synth.* **15**, 18–20.

[bb7] Krause, L., Herbst-Irmer, R., Sheldrick, G. M. & Stalke, D. (2015). *J. Appl. Cryst.* **48**, 3–10. 10.1107/S1600576714022985 PMC4453166 26089746

[bb8] Leigh, G. J. & Richards, R. L. (1982). In *Comprehensive Organometallic Chemistry: the synthesis, reactions, and structures of organometallic compounds*, edited by G. Wilkinson, F. G. A. Stone & E. W. Abel, Vol. 5, pp. 599–603. New York: Pergamon Press.

[bb9] Lide, D. R. (2002–2003). *Chem. Phys.* 83rd ed. Florida: CRC Press.

[bb10] Malosh, T. J., Shapley, J. R., Lawson, R. J., Hay, D. N. T. & Rohrabaugh, T. N. Jr (2013). *J. Organomet. Chem.* **745–746**, 98–105.

[bb11] Marcén, S., Jiménez, M. V., Dobrinovich, I. T., Lahoz, F. J., Oro, L. A., Ruiz, J. & Astruc, D. (2002). *Organometallics*, **21**, 326–330.

[bb12] Onderdelinden, A. L. & van der Ent, A. (1972). *Inorg. Chim. Acta*, **6**, 420–426.

[bb13] Porta, P., Powell, H. M., Mawby, R. J. & Venanzi, L. M. (1967). *J. Chem. Soc. A*, pp. 455–465.

[bb14] Sheldrick, G. M. (2008). *Acta Cryst.* A**64**, 112–122. 10.1107/S0108767307043930 18156677

[bb15] Sheldrick, G. M. (2015*a*). *Acta Cryst.* A**71**, 3–8.

[bb16] Sheldrick, G. M. (2015*b*). *Acta Cryst.* C**71**, 3–8.

[bb17] Winkhaus, G. & Singer, H. (1966). *Chem. Ber.* **99**, 3610–3618.

[bb18] Woodmansee, D. H., Müller, M.-A., Neuburger, M. & Pfaltz, A. (2010). *Chem. Sci.* **1**, 72–78.

